# Prevalence and characterization of forgoing care: comparison of two prospective multicentre cohorts between pre-COVID-19 era and a lockdown period

**DOI:** 10.1186/s13690-022-00797-3

**Published:** 2022-01-19

**Authors:** Delphine Douillet, Clémence Dupont, Noémie Leloup, Grégory Ménager, Maud Delori, Caroline Soulie, François Morin, Thomas Moumneh, Dominique Savary, Pierre-Marie Roy, Aurore Armand

**Affiliations:** 1 Emergency Department, Angers University Hospital, UNIV Angers, Angers, France; 2 UMR MitoVasc CNRS 6015 - INSERM 1083, Health Faculty, Angers, France; 3 Emergency Department, Le Mans Hospital, Le Mans, France; 4 Emergency Department, Cholet Hospital, Cholet, France; 5 EHESP, Irset, Inserm, UMR S1085, CAPTV CDC, University of Rennes, Rennes, France; 6 République des Savoirs- Lettres, Sciences, Philosophie - USR3608- ED540- ENS-PSL, Paris, France

**Keywords:** Forgoing care, healthcare renunciation, Emergency care, COVID-19, Coronavirus

## Abstract

**Background:**

Little is known about patients who forego healthcare, although it is an important provider of unfavorable health-related outcomes. Forgoing healthcare characterizes situations in which people do not initiate or interrupt a care process, even though they perceive the need for it, whether or not this need is medically proven. The aims of this study were to assess the prevalence and the determinants of patients who forego healthcare. The second aim was to compare the characteristics of patients who gave up healthcare during the French lockdown due to COVID-19.

**Methods:**

We conducted two multicenter cross-sectional studies in 2017 and 2020 carried out in French patients presenting to the emergency departments. Patients who gave their consent to participate were interviewed with a standardized questionnaire. It consisted of two parts: epidemiological characteristics and health care refusal. A third part concerning the renunciation of care during the COVID-19 period was added to the second study period.

**Results:**

A total of 1878 patients had completed the questionnaire during the interview with the physicians, 900 during the first period in 2017 (47.9%) and 978 (52.1%) during the second period. A total of 401/1878 patients reported not seeking care in the last 12 months (21.4% [95%CI: 19.5–23.3%]). In 2020, patients forewent care more during the confinement period than outside with different characteristics of the foregoing care populations.

**Conclusion:**

Forgoing care is common in a universal health care system such as France’s and increased during the pandemic. Key public health messages targeted at the reasons for not seeking care must now be disseminated in order to combat this.

**Supplementary Information:**

The online version contains supplementary material available at 10.1186/s13690-022-00797-3.

## Background

Forgoing healthcare, underuse of care or delayed care are providers of unfavorable health-related outcomes, such as higher severity of disease at the admission, decreased quality of life, increased hospitalization rates, and longer hospital stays [1–4]. And finally, foregoing health care lead to higher medical costs to treat advanced conditions or to deal with an emergency complication of an undiagnosed disease [2]. Forgoing healthcare characterizes situations in which people do not initiate or interrupt a care process, even though they perceive the need for it, whether or not this need is medically needed. The term is used interchangeably with unmet needs as both terms provide similar information. Forgoing care is currently assessed in the last 12 months of the patient’s life [5–8]. Studies on health care foregone are often used as a proxy for unmet need, whether due to financial or non-financial constraints, such as geographical distance, language, or system responsiveness. The prevalence of foregone care varies widely ranging from 3.1 to 25.6% [5, 6, 8–14].

Foregone care also depends on the organization of national healthcare systems. In France, since 1945, the health system has been grounded in a social conception promoting universalism and equality and is based on income and deducted from employees’ pay (around 7 to 20% of healthcare costs) [15]. Health costs seem to be well covered in France: 92% of these costs were reimbursed to French patients (compared with 87% in Germany, 79.6% in Belgium, 85% in Canada, and 88% in the United States) [16]. The remaining of health expenditures are financed by a system of private and/or public complementary health insurance and out-of-pocket payments accounting. However, healthcare foregone in France is estimated at between 13.7 and 25.6% [9, 13]. A final subgroup of patients is those with no health insurance coverage, such as first-time migrants and people on the margins of society (homeless, etc). Emergency departments are the first places where vulnerable populations can be provided care, who are also the populations most likely to foregone care [5, 17].

During the coronavirus disease 2019 (COVID-19) pandemic period, and even more so during the lockdowns, many hospitals and emergency departments saw a significant reduction in their admission numbers [18, 19]. The renunciation of care becomes a real issue during this period of coronavirus and a major concern for health caregivers. Anderson et al., reported a prevalence of 41% of respondents forwent medical care from March through mid-July 2020 in the United States [20].

However, to date, less is known about the prevalence of renunciation during the usual period compared to that during a pandemic and about the characteristics of the patients. Defining which patients are forgoing healthcare is a major public health issue, allowing targeted action to be taken on these populations or to transmit key messages to combat this dropout and its consequences.

The aims of this study were to assess the prevalence and the determinants of patients who forego healthcare. The second aim was to compare the characteristics of patients who gave up healthcare during the French lockdown due to COVID-19.

## Methods

### Design

We conducted two multicenter cross-sectional studies in 2017 and 2020 carried out in patients presenting to the emergency departments (ED). The two participating centers of EDs were urban centers, with an annual ED census around 70,000 visits per year. The ED of Angers University Hospital and Le Mans Hospital saw their activity fall by 30 and 36% respectively in 2020 compared to 2019 dur to COVID-19 pandemic. The STROBE (Strengthening the reporting of observational studies in epidemiology) checklist was followed.

### Participants and settings

During the inclusion periods of the study and after explaining the aims of the study and receiving their oral consent, each patient was interviewed by a physician to administer the questionnaire. The interviews were conducted on random days and at random times without prior selection of participants. If the patients did not speak French, a telephone translation platform was used. Minor patients, patients unable to give their consent for psychiatric or physical reasons, patients with severity criteria on arrival at the ED and requiring urgent resuscitation care were excluded.

### Survey administration

The first step was designing the questionnaire. A scientific committee drew up the questionnaire based on data from the literature and on the definition of health care forgoing. It included two main parts. The first part concerned the epidemiological characteristics of the population, citizenship, language spoken fluently, presence of housing, resources including the presence of a job, family situation, and reason for consultation. For the unemployed, the presence of other resources was collected (i.e., minimum social benefits, disability, unemployment, pensioner or absence of resources). And finally, medical care with the presence of a general practitioner (GP) and social insurance coverage were gathered. The second part sought to assess the renunciation of care. The question asked was: “Have you given up care (consultation with your general practitioner, specialist, emergency room, dentist) during the last 12 months when you felt the need?”. We have extended the definition to include foregoing prescribed care such as medications, lab tests or imaging. During the COVID-19 period an additional annex was proposed: we asked if the forgoing of healthcare had occurred during the lockdown period from March 16, 2020 to May 10, 2020 in France and reasons for this renunciation were sought in the form of different proposals (fear of being infected, fear of overloading the health care system, fear of infecting relatives, financial reasons, inability to travel, lack of knowledge of the health care system during the epidemic period, as well as with a free text). We asked them to specify whether this renunciation was related to the current epidemic. The presence of a current daily treatment and its possible modification during the COVID-19 epidemic as well as the use of self-medication were collected. Finally, information on whether the patient considered him/herself to be at risk of developing a severe form of COVID-19 and a self-assessment of the patients’ level of information about COVID-19 were gathered (Additional file 1).

### Outcome and endpoints

The primary aim was to assess the prevalence of foregone care in the population presenting to the ED encompassing both periods. The primary endpoint was the rate of patients who reported foregoing care in the past 12 months during individual interviews. The secondary endpoints were the characteristics of patient who forgone healthcare compared to patients who did not forego care.

Other endpoints were the comparison of patients who forego healthcare during the two-month lockdown COVID-19 period versus patients who forego healthcare outside the COVID-19 period. Then, the characteristics of the patients giving up care during the lockdown period were compared to those who didn’t foregone care. We did not include the 2017 population with the 2020 pandemic population because patients in 2017 could have foregone care during the lockdown period. The reasons for not seeking care were analyzed as well as some questions related to COVID-19 (Additional file 1).

### Statistical methods

Baseline characteristics were expressed as number (%) for categorical variables and mean (standard deviation [SD]) or median (interquartile range [IQR]) for continuous variables, depending on their distribution. Categorical data are reported as number and percentage. We performed comparisons between forgoing patients and non-forgoing patients by using paired student t-test or paired rank Wilcoxon test for continuous data, with determination of the mean difference and its 95% confidence interval (CI). For categorical variables, we calculated odds ratios (ORs) between those who have given up care and those who did not with their 95%CIs. All comparisons were 2-tailed, and a *p*-value less than 0.05 was required to reject the null hypothesis. Separate bivariate analyses were performed to determine the unadjusted association between foregoing healthcare and the following potential risk factors: age, sex, nationality, spoken language, housing, family situation, having children, supplemental coverage, followed by a GP, employment or not. A direct multivariable binary logistic regression model was built taking into account all this risk factors (*p*-value inferior or equal to 0.2 in univariate analysis or forced into the model). Multicollinearity was investigated using in first correlation matrix and in second tolerance and variation inflation parameters. With an exploratory aim and to assess the different foregoing characteristics, a hierarchical cluster analysis was made. The distance matrix (ward distance) then hierarchically clustered using complete-linkage clustering implemented in R was made. No imputation for missing data was performed. All data were analysed using R software (R Core Team, 2014, R: A language and environment for Statistics computing. R Foundation for Statistical Computing, Vienna, Austria). The forest plots were built using the “rmeta” and “forestplot” package in R.

### Ethics

The clinical research protocol was approved by the “Comité de Protection des Personnes Sud-Est VI (Clermont Ferrand)” under the ID-RCB No.: 2020-A01960–39. Prior to answering the questionnaire, an oral information and an information letter was given to the patient and his oral consent was collected. This study was reported to the Commission Nationale Informatique et Libertés (CNIL) and in the clinicaltrials website before the first inclusion (NCT04349163).

## Results

### Participants

**Fig. 1:**
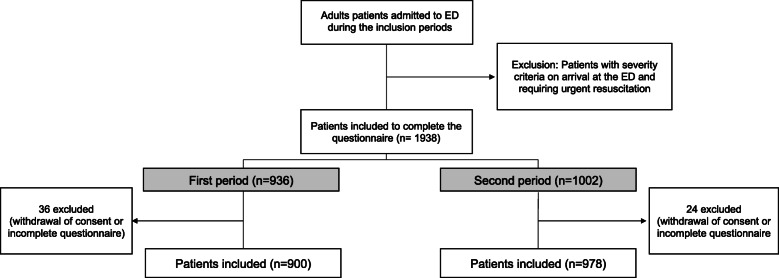
Study flow chart

A total of 1878 patients had completed the questionnaire during the interview with the physicians, 900/1878 during the first period in 2017 (47.9%) and 978/1878 (52.1%) during the second period (Fig. 1). Twenty-four patients were excluded because of uncomplete questionnaire. The populations were different on several criteria between 2017 and 2020 (Table 1). Each inclusion period lasted 4 months. The first period was carried out from March to June 2017 and the second period from July to October 2020. Of the participants, the average age was 47 years (± 20.9) and 923 were women (49.1%). The vast majority were of French nationality (94.3%) and spoke French fluently (99.3%). Slightly more than half were living with a partner (57.2%) and 68.4% had at least one child (Table 1). A total of 401/1878 patients reported not seeking care in the last 12 months (21.4% [95%CI: 19.5–23.3%]). In the first and second periods, this accounted for 208/900 (23.1% [95%CI: 20.4–26.0%]) and 193/978 (19.7% [95%CI: 17.3–22.4%]) patients respectively. There was no significant difference in the 12-month refusal rate between the two study phases (*p* = 0.17). The average rate of patients refusing care per month was 16.7 patients per month. The prevalence of foregoing healthcare in the different subgroups and according to the two periods is presented in the Additional file 2.

**Table 1:** Inclusion characteristics of the general population by inclusion phase

	Total *N* = 1878 (%)	Population phase 1 *N* = 900 (%)	Population phase 2 *N* = 978 (%)	*p*-value*
*Demographic characteristics*				
Age – years, median (± SD)	47 ± 20.9	50 ± 21.7	44 ± 19.9	0.06
18–25 years	284 (15.1)	122 (13.5)	162 (16.6)	0.07
26–59 years	944 (50.3)	422 (46.9)	522 (53.4)	< 0.05
> 60 years	649 (34.6)	356 (39.6)	293 (30.0)	< 0.05
Female sex	923 (49.1)	431 (47.9)	492 (50.3)	0.14
*Sociological data*				
Citizenship				0.12
French	1769 (94.2)	856 (95.1)	913 (93.4)	
Others	109 (5.8)	44 (4.9)	65 (6.6)	
Spoken French	1864 (99.3)	896 (99.6)	968 (99.0)	0.24
Lives in housing	1843 (98.1)	893 (96)	950 (97.1)	< 0.05
Family situation				< 0.05
In couple with child (ren)	883 (47)	427 (47.5)	456 (46.6)	
In couple without children	192 (10.2)	74 (8.2)	118 (12.1)	
Single with child (ren)	401 (21.4)	209 (23.2)	192 (19.6)	
Single without children	402 (21.4)	190 (21.1)	212 (21.7)	
Supplemental insurance coverage	1826 (97.2)	867 (96.3)	959 (98.1)	0.03
Followed by a GP	1769 (94.2)	864 (96)	905 (92.5)	< 0.05
Active worker	919 (48.9)	385 (42.8)	534 (54.6)	< 0.05

### Determinants of foregone health care

**Fig. 2:**
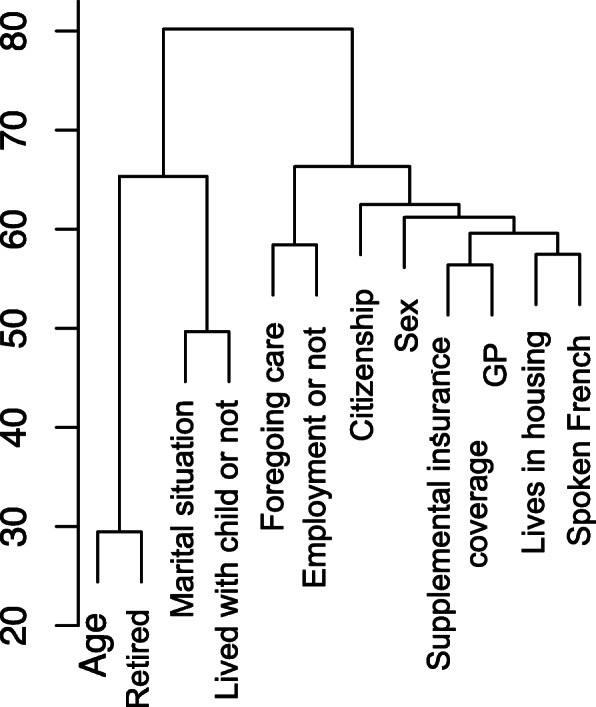
Hierarchical cluster-derived dendrogram displaying the co-clustering

The comparison of characteristics is summarized in Additional file 3 and Table 2. In the whole population, younger age, foreign nationality, living alone, lack of GP care were risk factors for not seeking care. Retired people gave up less care than other patients (*p* <  0.01). The absence of complementary insurance coverage was at the limit of significance. According to the dendrogram, we can distinguish 3 large homogeneous populations. Forgoing healthcare seems to reflect social characteristics (Fig. 2).

**Table 2:** Univariate and multivariable analysis of risk factors associated with not seeking care in the last 12 months

	Bivariate OR (95%CI)	*p*-value	Multivariate OR (95%)	*p*-value
Age – years	0.9 (0.6 to 0.9)	< 0.01	0.9 (0.7 to 0.9)	< 0.01
Female sex (vs male)	1.1 (0.8 to 1.3)	0.12	1.0 (0.7–1.2)	0.30
Foreign nationality (versus French)	9.0 (5.9 to 13.7)	< 0.01	1.4 (1.1 to 1.9)	< 0.01
Foreign spoken language (versus spoken French)	1.9 (0.3 to 3.3)	0.89	2.1 (0.5 to 2.3)	0.29
Housing (versus no housing)	1.3 (0.6 to 2.8)	0.53	1.2 (0.6 to 1.3)	0.73
Lives alone (versus in a couple)	1.4 (1.1 to 1.7)	< 0.01	1.3 (1.1 to 1.5)	0.05
Without children (versus with children)	1.6 (1.3 to 2.1)	< 0.01	1.1 (0.8 to 1.3)	0.55
Without supplemental coverage (versus with)	2.4 (1.3 to 4.2)	< 0.01	1.4 (0.7 to 2.1)	0.08
Without GP (versus followed by a GP)	2.8 (1.9 to 4.2)	< 0.01	1.6 (1.4 to 1.9)	< 0.01
Without employment (versus with employment)^a^	2.3 (1.8 to 2.9)	< 0.001	1.0 (0.3 to 1.3)	0.85
Retired (versus not retired)	0.3 (0.2 to 0.4)	< 0.001	0.5 (0.2 to 1.2)	< 0.01

*GP* General Practitioner

^a^ withdrawn retiree

### Comparison between the period before COVID-19 and during COVID-19

In 2020, 193/978 patients reported renouncing care in the last 12 months, 134/193 who gave up during the containment period (2 months) (69.4%) and the others outside this period (*n* = 59/193, 30.6%). Compared to patients who did not drop out in the second period, patients who dropped out in the COVID-19 period do not show any identifiable characteristics (Table 3).

**Table 3:** Characteristics of the patients who gave up care or not during the COVID-19 lockdown in the second phase of the study.

	Total *n* = 978 (%)	Population who has given up care during COVID-19 period *n* = 134 (%)	Population who did not forego care *n* = 844 (%)	*p*-Value
*Demographic characteristics*				
Age – years, median (± SD)	44 ± 19.9	41 ± 18.2	45 ± 20.1	0.72
Female sex	492 (50.3)	79 (59.0)	413 (48.9)	0.07
*Sociological data*				
Citizenship				
French	913 (93.4)	121 (90.3)	792 (93.8)	0.12
Others	65 (6.6)	13 (9.7)	52 (6.2)	
Spoken French	968 (99.0)	133 (99.3)	835 (98.9)	0.73
Lives in housing	950 (97.1)	134 (100)	816 (96.7)	0.31
Family situation				
In couple	575 (58.8)	71 (53.0)	504 (59.7)	0.14
Single	403 (41.2)	63 (47.0)	340 (40.3)	
With child (ren)	648 (66.3)	85 (63.4)	563 (66.7)	0.45
Without child (ren)	330 (33.7)	49 (36.6)	281 (33.3)	
Supplemental insurance coverage	959 (98.1)	129 (96.3)	830 (98.3)	0.10
Followed by a GP	905 (92.5)	121 (90.3)	784 (92.9)	0.28
Occupational status				0.88
Active worker	534 (54.6)	74 (55.2)	460 (54.5)	ref
Disabled worker	60 (6.1)	14 (10.4)	46 (5.5)	
Unemployment or social minima	72 (7.4)	9 (6.7)	63 (7.5)	
No resources	87 (8.9)	13 (9.7)	74 (8.8)	
Retirement	225 (23.0)	24 (17.9)	201 (23.8)	
Long-term treatment	367 (37.5)	53 (39.6)	314 (37.2)	0.09

*GP* General Practitioner

**Fig. 3:**
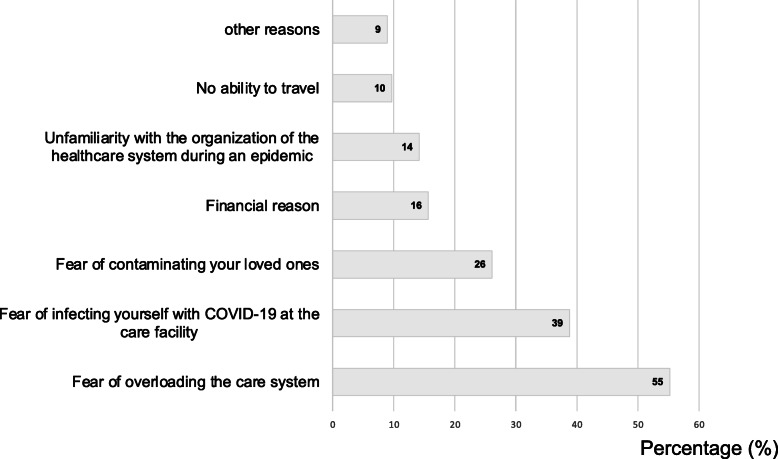
Reasons for not seeking care during the COVID-19 period

The reasons for not coming forward were mainly fear of overloading the emergency department (*n* = 74/134, 55%), fear of becoming infected with COVID-19 (*n* = 52/134, 39%) and fear of infecting relatives (*n* = 35/134, 26%) (Fig. 3). Five percent of participants did not take any prescribed medications (*n* = 49/978, 5%), biological (*n* = 45/978, 5%) or radiological (40/978, 4%) treatment during the second period. Of the population who forewent care during the lock-in period, 53/134 (40%) were on long-term treatment, 30/134 were self-medicating (22%). Of the population who forewent care, 32 patients thought they might have a severe form of COVID-19 if they were infected (24%). Regarding their level of information about COVID-19 on a Likert scale of 1 to 10, the average was 7.2 (±1.4) in the renouncing population versus 7.5 (±1.5) in the non-renouncing population.

## Discussion

The prevalence of foregoing healthcare remains high among patients addressing to the EDs in France: slightly less than a quarter of people have already given up care when they felt the need to do so in the last 12 months. This rate is globally stable between 2017 and 2020. However, patients who visited EDs in 2017 and 2020 during the pandemic did not have the same characteristics. Patients who forego healthcare care was higher during the lockdown period for various explanatory reasons.

The prevalence of healthcare renunciation was 21.4% [95%CI: 19.5–23.3%] in line with previous studies in France and Switzerland among adult populations by Baggio et al. [27.3%], Guessous et al. [14.0%] and Desprès et al. [15.4%] [14, 21, 22]. However, the comparison of the rates of renunciation of care between the studies is difficult because of the heterogeneity of the populations questioned. In the other studies, the participants were a selected sub-group of these populations [21]. This study had broad inclusion criteria although it was conducted in ED. EDs are a place of admission for precarious patients and people who have given up on care and arrive late in the care system. Deprived patients from lower socio-economic geographical areas have been shown to access more emergency care [23, 24]. However, the foregone rate is average and appears to be broadly stable although there are only two measures over time (i.e., 2017 and 2020). The evolution of this proportion of people foregoing health care was described as stable also by Chaupin and Guillot et al. [25] and increasing in the Swiss study by Guessous et al. [14] The Subjective Unmet Need (SUN) is a synonymous concept proposed as an overall indicator of the health care system and especially of its access [11]. Regular assessment of this item in a population is therefore essential as it reflects the real difficulties in the care pathways. Since the SARS-CoV-2 pandemic, a decrease in ED visits by non-COVID-19 patients, a sharp decrease in the incidence of cardiac events, and a decrease in visits to treating physicians have quickly led to fears of a massive renunciation of care among non-COVID-19 infected patients [19, 26, 27]. In this study, as in others published recently, the rate of renunciation of care has increased during the lockdown months [20, 28]. However, if the pandemic is sustained, adults may miss opportunities to manage chronic diseases, follow up on their routine screenings, and detect new diseases early, which could worsen outcomes. Spillover effects of the COVID-19 pandemic could drive long-term health consequences for non-COVID-19 patients.

Out of the COVID-19 period, specific social determinants were significantly associated with a higher rate of refusal of care. These were young age, foreign nationality, living alone and the absence of a general practitioner were risk factors for not seeking care. These items are consistent with the literature [5, 14]. The absence of complementary coverage was at the limit of significance. This is a criterion frequently found to be associated with not seeking care [29, 30]. Knowing the populations at risk of dropping out can allow for targeted public health policy, however, in this study we found that the profile of patients who forego healthcare changed during the pandemic period. We do not find any difference with the characteristics of the general population regarding the items studied. The change in the profile of patients giving up care was also noted in the study by Czeiler et al [28] The reasons for foregoing care might be different, with the majority of them relating to the safety of health facilities (fear of infecting themselves and their relatives) and the fear of overloading the health care system. These two obstacles that can be overcame if the health system has the capacity to be resilient: differentiation of the reception of COVID-19 and non-COVID-19 patients and the capacity to deal with the wave of the pandemic in addition to the usual care. The public health messages to be disseminated must in any case take account of these fears in order to reassure the population and limit this foregoing healthcare. The foregoing healthcare measures the fact of not being able to seek care when one feels the need for it, but also the foregoing healthcare in the context of a refusal of care. In the context of the pandemic, where the health situation has had a strong impact on the daily life of each person, it is possible that refusal of care has become more important. It would be interesting in future studies to evaluate more precisely the reasons for refusal of care and to repeat this evaluation on a regular basis. Finally, forgoing care may be just one part of a more comprehensive approach to changing consumer health behavior since the pandemic.

### Limitations

The findings in this report are subject to limitations. Firstly, the data on renunciation of care was declarative. Participants may have under- or overreported some problems. We were not able to verify the absence of care use during the 12 previous months. This limitation also included a problem of adaptive preferences. The subjective evaluation of the healthcare needs of poor participants could be lowered because they prioritize basic necessities rather than health [14]. Therefore, the prevalence of healthcare renunciation may be underestimated. The study was conducted in a population that may have foregone care but presents itself in an ED. This study did not assess the proportion of tests, consultations, or prescribed medications that were not followed up after presentation to the ED. It would be interesting to evaluate this in a new prospective study. This does not allow us to extrapolate to the entire population that may have given up. Furthermore, we were not able to establish a collection of patients not included in the study in order to assess potential selection bias. Thus, the results should be interpreted with caution. Further studies are needed to assess this issue. Finally, the use of digital health has not been evaluated. It is possible that some patients report not having consulted but still benefited from tele-medicine assessment.

## Conclusions

To conclude, forgoing care is common in a universal health care system such as France’s and increased during the pandemic. Meanwhile, efforts should be made to prevent high-risk populations from forgoing care. Targeting patients most at risk of not seeking care and providing reassurance about safe, quality care during a pandemic is now a major public health issue.

## Supplementary Information

**Additional file 1.** Patient questionnaire.

**Additional file 2.** Prevalence of foregoing healthcare in the different subgroups and according to the two periods.

**Additional file 3.** Baseline characteristics according to whether or not they have given up care in the last 12 months.

## Data Availability

Available on special request from the investigators.

## Abbreviations

COVID-19: Coronavirus disease 2019
ED: Emergency Department
GP: General Practitioner
IQR: Interquartile Range
OR: Odds Ratio
95%CI: 95% confidence interval
