# Supplementary material for: Prevalence and characterization of forgoing care: comparison of two prospective multicentre cohorts between pre-COVID-19 era and a lockdown period
Source: Arch Public Health. 2022 Jan 19;80:32. doi: 10.1186/s13690-022-00797-3 (PMC8766360; doi:10.1186/s13690-022-00797-3)
Supplement: Supplementary file 1 — Additional file 1. Patient questionnaire. [file 13690_2022_797_MOESM1_ESM.docx]

Supplemental Digital Content 2. Patient questionnaire.

**PATIENT:**

Weight: ............ kgs Height: ............ cm

Nationality: □ French □ Other: .......................

Are you fluent in French? □ Yes □ No

Reason for visiting the UAS: .................................

**PERSONAL AND FAMILY SITUATION:**

Do you have housing? □ Yes □ No

Are you living: □ In a relationship □ Alone

Do you have social security coverage? □ Yes □ No

Do you have any children? □ Yes □ No

Do you have a primary care physician? □ Yes □ No

**RESOURCES:**

Do you work? □ Yes □ No

If yes, what work do you do? ......................

Do you have any other types of resources?

□ Social minima □ Disability □ Unemployment □ No resources

**FOREGONE CARE:**

Did you, during the period of confinement (from 16/03/2020 to 10/05/2020), when you felt the need, give up consulting a health professional (doctor, dentist, optician)? □ Yes □ No

If yes:

- What is (are) the reason(s) you gave up care?

□ Fear of infecting yourself with COVID-19 at the care facility

□ Fear of overloading the care system

□ Fear of contaminating your loved ones

□ Financial reason

□ No ability to travel

□ Unfamiliarity with the organization of the healthcare system during an epidemic

□ Other: ..........

Did you during the lockdown period (3/16/2020 to 5/10/2020) forgo any physician-prescribed care?

- Medication: □ Yes □ No

- Laboratory tests (blood work, urine tests...): □ Yes □ No

- Imaging exam (x-ray, CT scan, MRI...): □ Yes □ No

Are you taking any background treatment? □ Yes □ No

If yes, did you change it on your own without medical advice with the COVID outbreak? □ Yes □ No

Did you self-medicate? □ Yes □ No

**COVID-19:**

Do you consider yourself at risk for a severe form of COVID-19? □ Yes □ No

How would you rate your personal level of information about COVID-19? ............/10

(Put a score between 0 and 10, knowing 0 = no information and 10 = maximum information)
