# Supplementary material for: Prevalence and characterization of forgoing care: comparison of two prospective multicentre cohorts between pre-COVID-19 era and a lockdown period
Source: Arch Public Health. 2022 Jan 19;80:32. doi: 10.1186/s13690-022-00797-3 (PMC8766360; doi:10.1186/s13690-022-00797-3)
Supplement: Supplementary file 2 — Additional file 2. Prevalence of foregoing healthcare in the different subgroups and according to the two periods. [file 13690_2022_797_MOESM2_ESM.pptx]

## Slide 1
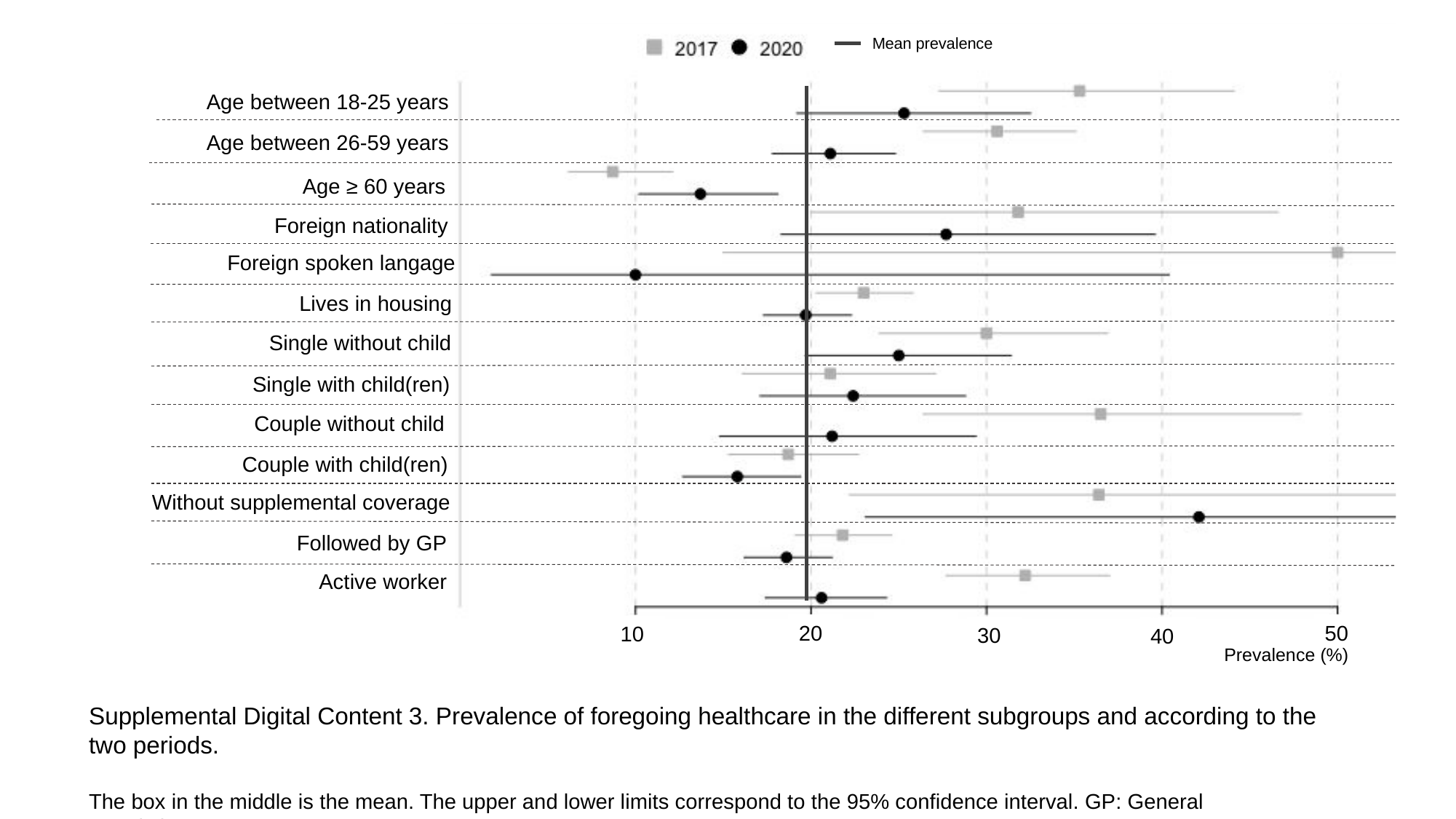

Mean prevalence
Age between 18-25 years
Age between 26-59 years
Age ≥ 60 years
Foreign nationality
Foreign spoken langage
Lives in housing
Single without child
Single with child(ren)
Couple without child
Couple with child(ren)
Without supplemental coverage
Followed by GP
Active worker
50
20
10
30
40
Prevalence (%)
Supplemental Digital Content 3. Prevalence of foregoing healthcare in the different subgroups and according to the two periods.
The box in the middle is the mean. The upper and lower limits correspond to the 95% confidence interval. GP: General Practictionner.
