# Supplementary material for: Prevalence and characterization of forgoing care: comparison of two prospective multicentre cohorts between pre-COVID-19 era and a lockdown period
Source: Arch Public Health. 2022 Jan 19;80:32. doi: 10.1186/s13690-022-00797-3 (PMC8766360; doi:10.1186/s13690-022-00797-3)
Supplement: Supplementary file 3 — Additional file 3. Baseline characteristics according to whether or not they have given up care in the last 12 months. [file 13690_2022_797_MOESM3_ESM.docx]

| Supplemental Digital Content 4. Baseline characteristics according to whether or not they have given up care in the last 12 months. | | | | |
| --- | --- | --- | --- | --- |
|  | Total  n= 1878 (%) | Population who have given up care  n= 401 (%) | Population who did not forego care  n= 1477 (%) | P-Value |
| *Demographic characteristics* |  |  |  |  |
| Age – years, median (± SD) | 47 ± 20.9 | 37 ± 17.5 | 49 ± 21.3 | <0.01 |
| Female sex | 983 (52.3) | 214 (53.4) | 769 (52.1) | 0.12 |
| *Sociological data* |  |  |  |  |
| Citizenship |  |  |  | 0.08 |
| French | 1771 (94.3) | 329 (82.0) | 1442 (97.6) |  |
| Others | 107 (5.7) | 72 (18.0) | 35 (2.4) |  |
| Spoken French | 1863 (99.2) | 398 (99.3) | 1465 (99.2) | 0.89 |
| Lives in housing | 1843 (98.1) | 392 (97.8) | 1451 (98.2) | 0.53 |
| Family situation |  |  |  |  |
| In couple | 1075 (57.2) | 204 (50.9) | 871 (59.0) | <0.01 |
| Single | 803 (42.7) | 197 (49.1) | 606 (41.0) |  |
| With child(ren) | 1284 (68.4) | 239 (59.6) | 1045 (70.8) | <0.01 |
| Without child(ren) | 594 (31.6) | 162 (40.4) | 432 (29.2) |  |
| Supplemental insurance coverage | 1826 (97.2) | 381 (95.0) | 1445 (97.8) | <0.01 |
| Followed by a GP | 1769 (94.2) | 356 (88.8) | 1413 (95.7) | <0.01 |
| Occupational status |  |  |  | <0.01 |
| Active worker | 940 (50.1) | 224 (55.9) | 716 (48.5) | ref |
| Disabled worker | 108 (5.8) | 46 (11.5) | 62 (4.2) |  |
| Unemployment or social minima | 131 (7.0) | 31 (7.7) | 100 (6.8) |  |
| No resources | 151 (8.0) | 47 (11.7) | 104 (7.0) |  |
| Retirement | 548 (29.2) | 53 (13.2) | 495 (33.5) |  |

GP : General Practitioner
